# MiR-219-5p Inhibits the Growth and Metastasis of Malignant Melanoma by Targeting BCL-2

**DOI:** 10.1155/2017/9032502

**Published:** 2017-08-13

**Authors:** Jianwen Long, Qiqige Menggen, Qimige Wuren, Quan Shi, Xianming Pi

**Affiliations:** ^1^Department of Dermatology, The First Clinical Medicine School, Hubei University of Chinese Medicine, Wuhan, China; ^2^Department of Dermatology, Hubei Provincial Hospital of Traditional Chinese Medicine, Wuhan, China; ^3^Department of Dermatology, Hubei Provincial Academy of Traditional Chinese Medicine, Wuhan, China; ^4^Department of Dermatology, Mongolian Medicine Hospital, Bortala Mongol Autonomous Prefecture, China

## Abstract

Malignant melanoma is a very dangerous tumor which is resistant to conventional therapy. MicroRNA exerts a vital function in promoting or inhibiting tumor development. The research has investigated the expression and function of miR-219-5p in melanoma. As a result, miR-219-5p expression was distinctly reduced in melanoma tissues and cell lines and was negatively correlated with Bcl-2 protein level in melanoma. Patients with low miR-219-5p level represented obviously a low overall survival in comparison with patients with high miR-219-5p level. The upregulation of miR-219-5p inhibited melanoma growth and metastasis and strengthened melanoma cells chemosensitivity by targeting Bcl-2. Therefore, the modulation of miR-219-5p expression may be a novel treatment strategy in melanoma.

## 1. Introduction

Malignant melanoma developing from melanocytes has shown significantly clinicopathological characteristics of high invasion, high metastasis, and high mortality [1, 2]. Although remarkable advances have been achieved in the treatment of tumors recently [3], the prognosis of metastatic melanoma remains still unsatisfied [4]. The etiology of melanoma is very complicated, involving cell differentiation, cell proliferation, cell apoptosis, and so on [5]. With the development of bioinformatics, new molecular targets have been discovered and implemented at the diagnosis and treatment of tumors [6].

MicroRNAs (miRNAs) are a kind of small 15–22-nucleotide noncoding RNA [7]. Many researches point out that miRNA is correlated with tumor growth and metastasis via acting as protumor or antitumor factors in a few neoplasms [8]. Meanwhile, some miRNAs are helpful to the occurrence, development, growth, metastasis, and chemotherapy resistance of melanoma through binding to the targeting mRNA [9–11]. Abnormal expression of miR-219-5p has been ascertained in some malignant tumors, and miR-219-5p has been identified to exert tumor suppressor function in a procession of different tumors [12, 13]. The expression and function of miR-219-5p have not been elucidated in melanoma up to date. The research aims to determine miR 219-5p level and investigate the specific effect of miR-219-5p on the development of melanoma.

## 2. Materials and Methods

### 2.1. Patients and Melanoma Samples

42 of melanoma tissues and 20 nevi tissues were acquired from the Hubei provincial hospital of Traditional Chinese Medicine by resection and were stored in liquid nitrogen. Patients did not receive chemotherapy or radiation therapy before resection.

This research was approved by the ethical committee of the Hubei Provincial Hospital of Traditional Chinese Medicine, and written informed consent was obtained from individuals with melanoma. These clinical features of the individuals with melanoma are represented in Table 1.

### 2.2. Cell Line and Reagents

A375, WM35, SK-MEL-5, and SK-MEL-2 cell lines were obtained from cell bank of Wuhan university and incubated in DMEM added with 10% fetal bovine serum, 100 U/mL penicillin, and 100 *μ*g/mL streptomycin (Gibco, USA) in an atmosphere of 5% CO2 at 37°C. Human epidermal melanocytes (HEMa-LP) were gained from Cascade biologists, UK, and were incubated in HMGS-2 medium in the same condition as described in melanoma cell lines.

### 2.3. Plasmid Transfection

MiR-219-5p mimics and miR-control and pcDNA3.1 vector encoding Bcl-2 were obtained from GeneCopoeia. Transfection was applied with Lipofectamine 2000 reagent (Invitrogen) on the basis of the protocol description.

### 2.4. Qualitative RT-PCR

Total RNA was extracted from tissues and cells through the TRIzol reagent (Invitrogen) on the basis of the reagent kit protocol. The cDNA was synthesized and amplified using the TaqMan miRNA reverse transcription kit. The mRNA levels of miR-219-5p and U6 were determined by qRT-PCR using TaqMan human MiRNA assay kit. The 2 − ΔΔCT method was utilized to measure the relative fold difference, MiR-219-5p: (F) 5′-ACACTCCAGCTGGGTGATTGTCCAAACGCAAT-3′ and (R) 5′-CTCAACTGGTGTCGTGGA-3′, U6: (F) 5′-CTCGCTTCGGCAGCACA-3′ and (R) 5′-AACGCTTCACGAATTTGCGT-3′.

### 2.5. CCK-8 Assay

Melanoma cells proliferation activity was detected by CCK-8 assay according to the protocol's instruction. A375 cells after transfection are cultured in 96-well plates with the same conditions. At 12, 24, 48, and 72 h, 90 *μ*l fresh culture media and 10 *μ*l CCK-8 solutions were added to each sample. Subsequently, transfected melanoma cell was incubated at 37°C for 2 h, and the value was examined by a microplate reader at 450 nanometers.

### 2.6. Analysis of Apoptosis

At 48 hours after transfection, 2 × 10^5^ A375 cells were cultured in six-well plates. The cells were washed and resuspended in buffer. The cell apoptosis was detected by flow cytometric assay with Annexin V-FITC and PI staining.

### 2.7. Transwell Chamber Assay

A375 cells were cultured for 48 h after transfection. Transwell assay was applied to determine the cell invasion activity. Transwell chamber membrane was coated with Matrigel. 2 × 10^5^ cells were added in the upside chambers with serum-free medium. DMEM with 10% FBS was put into the downside chamber. After incubating for 48 h, swabs were utilized to wipe off the cells in the upside chamber; cells in the downside chamber were fixed and stained with 0.5% crystal violet. Cells count was performed using a microscope.

### 2.8. Wound Healing Assay

Cells after transfection were cultured in appropriate condition for 48 hours. Wounds were created by plastic scriber on the cell monolayer. Cells were then washed and incubated in DMEM with 10% FBS for 48 h. The migration activity was recorded using a microscope at 0 and 48 h and determined by subtracting the final wound width from the initial wound width

### 2.9. Western Blot Assay

Cells were lysed, protease inhibitors were added to the lysates and cell lysate was centrifuged at 12000 Rpm at 4°C. The protein concentration was examined by the BCA kit (Pierce, USA). The protein was separated using 10% SDS-PAGE assay and moved to a PVDF membrane. Antibodies listed below were applied to determine the protein expression: Bcl-2 (1 : 500, Santa Cruz, USA), cleaved caspase 3 (1 : 500, Abcam, USA), and cleaved caspase 9 (1 : 500, Abcam, USA). Anti-*β*-actin (1 : 2000, Sigma-Aldrich, USA). Horseradish peroxidase-conjugated secondary antibody (1 : 1000, Abcam, USA). An ECL chemiluminescent kit (Millipore, USA) was applied to measure the bands.

### 2.10. Luciferase Reporter Assay

The 3′-UTR sequence of Bcl-2 or the mutated sequence was cloned into pMIR-REPORT vectors. 50 nM miR-219-5p mimics or corresponding control was transfected together with a reporter plasmid and 1 ng pRL-SV40 (Promega, USA) into the A375 cells. After 48 h, the active Luciferase level was determined by a Luciferase Reporter Assay (Promega, USA).

### 2.11. Chemosensitivity Assay

48 h after transfection, A375 cells were incubated in fresh medium and then added to 96-well plates. Various doses of cisplatin or 5-FU were added to the cell culture for 72 h. Next, CCK-8 assay was applied to detect the cell survival.

### 2.12. Tumor Growth In Vivo

After transfection, A375 cells were transplanted into nude mice (25–30 g, six-week-old, *n* = 6), mice were sacrificed on 5 d, 10 d, 15 d, 20 d, and 25 d, and the weight and volume of tumors were measured and recorded.

### 2.13. Statistical Analysis

The SPSS 15.0 was applied for statistical analyses, including *t*-test, one-way analysis of variance, Fisher exact probability test, the Kaplan-Meier plot, and Pearson correlation analyses. All of the results were recorded as mean ± standard deviation. *P* < 0.05 was believed as statistically significant.

## 3. Results

### 3.1. MiR-219-5p Is Downregulated in Melanoma and Correlated with Unfavorable Prognosis in Individuals with Malignant Melanoma

**Figure 1:**
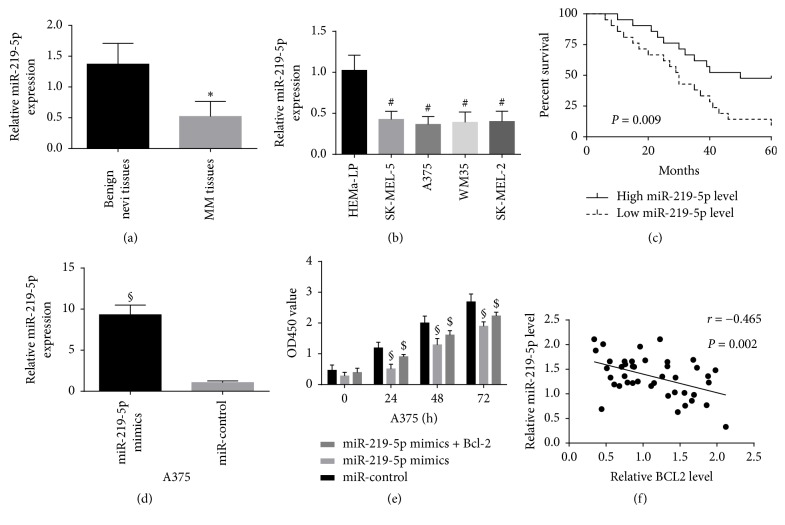
The expressions of miR-219-5p were detected in melanoma tissues and cells.* Notes*. (a and b) miR-219-5p was downregulated in melanoma samples and cells. (c) Low miR-219-5p level was correlated with decreased overall survival. (d and e) The transfection of miR-219-5p elevated the level of miR-219-5p in A375 cells and inhibited the proliferation of A375 cells. (f) An obviously inverse association was confirmed between miR-219-5p level and Bcl-2 protein level in melanoma tissues. ^#^*P* < 0.05 compared with HEMa-LP cell; ^*∗*^*P* < 0.05 compared with benign nevi tissue; ^§^*P* < 0.05 compared with miR-control; ^$^*P* < 0.05 compared with miR-219-5p mimics.

The miR-219-5p levels in 42 of melanoma tissues and 20 of benign nevi tissues were investigated using qRT-PCR. Our results manifested that the miR-219-5p levels were obviously decreased in melanoma samples in comparison with benign nevi tissues (Figure 1(a)). The miR-219-5p levels in A375 cells, WM35 cells, SK-MEL-2 cells, and SK-MEL-5 cells were distinctly downregulated compared with HEMa-LP cells (Figure 1(b)). Patients with melanoma were divided into high miR-219-5p expression group (*n* = 21) and low miR-219-5p expression group (*n* = 21) according to the miR-219-5p average level. The correlation between the miR-219-5p expression and the clinical features of individuals with melanoma was investigated. There was no obvious correlation between the age and sex of patients and miR-219-5p expression, but the low expression of miR-219-5p was distinctly related to the TNM stage and distant migration of malignant melanoma as described as Table 1. Besides, low miR-219-5p level was correlated with decreased overall survival (Figure 1(c)). The results manifested that downregulation of miR-219-5p was closely correlated with the progression of melanoma. The transfection of miR-219-5p elevated the level of miR-219-5p in A375 cells and inhibited the proliferation of A375 cells, but the cotransfection of Bcl-2 weakened the inhibition function induced by miR-219-5p in A375 cells (Figures 1(d) and 1(e)). Pearson's correlation test was applied to detect the relationship between miR-219-5p level and Bcl-2 level in melanoma samples. An obviously inverse association was confirmed between miR-219-5p level and Bcl-2 protein level in melanoma tissues (Figure 1(f)).

### 3.2.  Upregulation of miR-219-5p Inhibited A375 Cells Proliferation and Enhanced Apoptosis of A375 Cells

**Figure 2:**
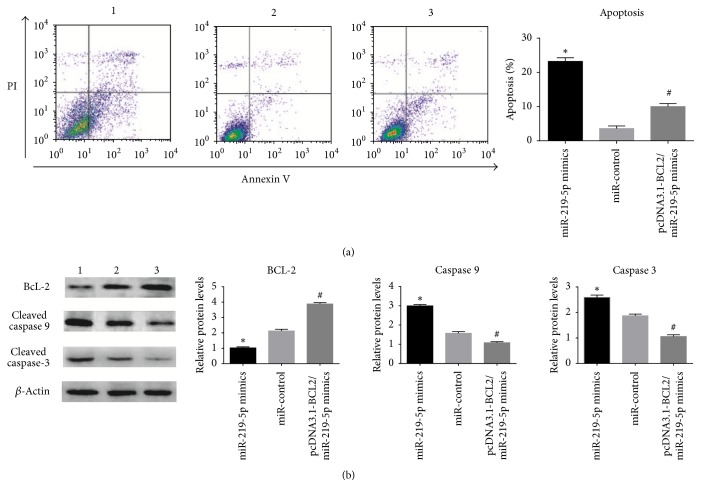
Flow cytometric analyses and Western blot were utilized to study the actions of miR-219-5p on A375 cells.* Notes*. (a) miR-219-5p promoted A375 cells apoptosis. (b) miR-219-5p decreased BCL2 expression and increased cleaved caspase 3 and cleaved caspase 9 expression in A375 cells. The cotransfection of Bcl-2 partially crippled the inhibition function of miR-219-5p.* Note*. 1: miR-219-5p mimics; 2: miR-control; 3: miR-219-5p mimics/pcDNA3.1 vector containing BCL2. ^*∗*^*P* < 0.01 versus miR-control; ^#^*P* < 0.01 versus miR-219-5p mimics.

A375 cells transfected with miR-219-5p were subjected to proliferation assay and flow cytometric analysis. The results as illustrated in Figure 2 confirmed that high expression of miR-219-5p suppressed the A375 cells growth and promoted cell apoptosis; however, the cotransfection of Bcl-2 inversed partially the proliferation and apoptosis of A375 cells induced by miR-219-5p. Subsequently, we detected the protein expression related to apoptosis; indeed, the decreased level of Bcl-2 and the increased level of cleaved caspase 3 and cleaved caspase 9 were identified in miR-219-5p group compared with miR-control group. However, the effect of miR-219-5p on A375 cells was inhibited by the cotransfection of Bcl-2.

### 3.3. Upregulation of miR-219-5p Inhibited the Invasion and Migration of A375 Cells

**Figure 3:**
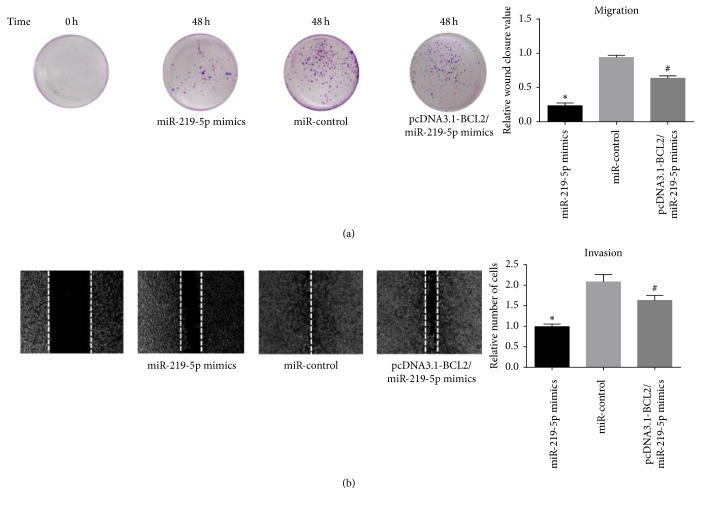
Upregulation of miR-219-5p suppressed A375 cells metastasis.* Note*. (a) Upregulation of miR-219-5p distinctly inhibited the A375 cells metastasis. (b) The cotransfection of Bcl-2 obviously alleviated the inhibition of miR-219-5p in A375 cells metastasis.^*∗*^*P* < 0.05, compared with miR-control group. ^#^*P* < 0.05, compared with miR-219-5p group.

Metastasis is the important causation of death associated with malignant melanoma; transwell assay and wound healing assay were performed to determine the influence of miR-219-5p on A375 cells metastasis (Figure 3). Ectopic expression of miR-219-5p obviously inhibited the metastasis of A375 cells. But the cotransfection of Bcl-2 weakened the function of miR-219-5p on A375 cells.

### 3.4. BCL-2 mRNA Was Confirmed as a Binding Target of miR-219-5p

**Figure 4:**
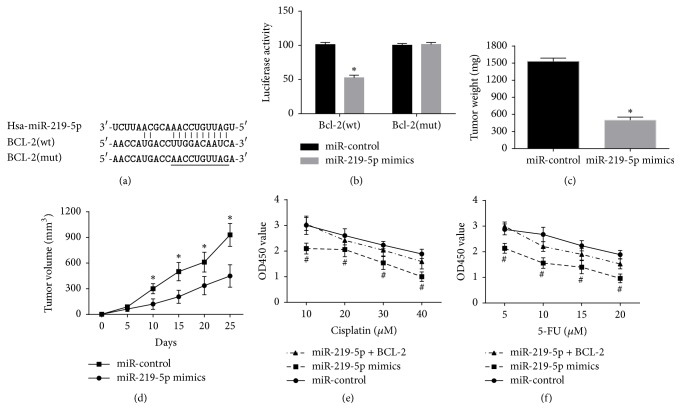
BCL-2 was a functional targeting mRNA of miR-219-5p on A375 cells.* Note*. (a) miR-219-5p and its predicted binding sequence for WT in BCL-2. (b) MiR-219-5p decreased Luciferase Reporter activities of Bcl-2 in wild type but had no significant impact on that of Bcl-2 in mutated type. (c) The transfection of miR-219-5p decreased the tumor volume. (d) The transfection of miR-219-5p reduced the tumor weight. (e and f) The transfection of miR-219-5p reinforced chemosensitivity to cisplatin or 5-FU treatment in A375 cells, but cotransfection of Bcl-2 weakened the function of miR-219-5p on A375 cell proliferation. ^*∗*^*P* < 0.05, compared with miR-control. ^#^*P* < 0.05, compared with miR-219-5p group.

Two important databases TargetScan 7.0 and miRanda were used for searching for the underlying downstream targets of miR-219-5p in melanoma cells. Bcl-2 was considered as a possible target for further study. The Luciferase Reporter analysis identified that 3-UTR of Bcl-2 was a downstream binding target of miR-219-5p as illustrated as Figure 4(a). The results manifested miR-219-5p obviously inhibited Luciferase activity in wild type but did not affect that in mutated type (Figure 4(b)).

### 3.5. miR-219-5p Inhibited Melanoma Growth in an Animal Experiment and Enhanced Chemosensitivity in A375 Cells

The role of miR-219-5p in melanoma was identified in nude mice models. The volume of tumors was obviously reduced in the miR-219-5p mimics group in comparison with those in the miR-control group (Figure 4(d)). The final weight of tumors was distinctly decreased in miR-219-5p mimics group compared with miR-control group (Figure 4(c)). Accordingly, the inhibition function of miR-219-5p on the melanoma growth was identified in vivo. Meanwhile, the transfection of miR-219-5p reinforced chemosensitivity to cisplatin or 5-FU treatment of A375 cells, but cotransfection of BCL-2 weakened this function of miR-219-5p on A375 cells.

## 4. Discussion

Tumor development is a complicated evolution and regulated by protumor and antitumor elements [14]. More and more researches have identified that microRNAs exert important function in tumorigenesis via modulating the targeting gene expression, including cell proliferation, differentiation, apoptosis, and metastasis [15, 16], MiR-219-5p has been confirmed to be expressed abnormally and play antitumor roles in a few tumors. For example, miR-219-5p inhibited the growth and metastasis of gastric cancer cells via targeting LRH-1 [17], and miR-219-5p exerted a antitumor function in colon cancer via binding to Sall4 [12]; however, the expression and effect of miR-219-5p in melanoma still remained unclear. In this article, the miR-219-5p level in melanoma tissues and cell lines was found to be downregulated. The low miR-219-5p level was obviously related to the malignant clinical features of patients with melanoma. Furtherly, it was identified that miR-219-5p suppressed the A375 cells proliferation and metastasis and promoted A375 cells apoptosis. Ectopic expression of miR-219-5p inhibited melanoma growth in vivo. These data demonstrated that miR-219-5p exerted a tumor suppressor function in melanoma. To investigate the peculiar regulatory mechanism of miR-219-5p on the development of melanoma, we retrieved two important databases, TargetScan 7.0 and miRanda, and found Bcl-2 may be an important downstream targeting gene, and we confirmed that the Bcl-2 protein level was significantly inversely correlated with the miR-219-5p level in tumor tissues. Furthermore, the Luciferase Reporter analysis revealed BCL-2 mRNA was exactly a binding target of miR-219-5p.

Bcl-2 gene, an important antiapoptotic gene, influences the intrinsic apoptosis pathway [18, 19]. Meanwhile, Bcl-2 can promote tumor invasion and metastasis [20]. Inhibition of Bcl-2 expression has been believed as an important treatment strategy for various cancers [21]. The following assays identified that the Bcl-2 level was significantly reduced, and the cleaved caspase 3 and 9 level were elevated in miR-219-5p group compared with the control group. Cotransfection of Bcl-2 recovered partially the proliferation and metastasis function of A375 cells inhibited by miR-219-5p and suppressed the apoptosis of A375 cells. These results showed that the impact of miR-219-5p on melanoma development was made by in part targeting the Bcl-2 mRNA.

In briefly, the research confirms that miR-219-5p exerts an antitumor function via partially regulating the Bcl-2 protein expression. The mediation of miR-219-5p may be a new and crucial scheme for molecular treatment of melanoma.

## Figures and Tables

**Table 1:** Relationship between the clinical feature and miR-219-5p level in patients with melanoma.

Characteristics	Number of patients	miR-219-5p		*P* value
	*N* = 42	High expression	Low expression	
Age (years)				
≦40	22	8	14	0.534
>40	20	10	10	
Sex				
Male	20	8	12	0.354
Female	22	13	9	
TNM stage				
I + II	14	10	4	0.019^*∗*^
III + IV	28	8	20	
Distant metastasis				
No	16	10	6	0.029^*∗*^
Yes	26	7	19	

*∗* refers to *P* < 0.05.
